# Effect of Zr and Ti Addition and Aging Treatment on the Microstructure and Tensile Properties of Al-2%Cu-Based Alloys

**DOI:** 10.3390/ma15134511

**Published:** 2022-06-27

**Authors:** Ehab Samuel, Ahmed M. Nabawy, Agnes M. Samuel, Herbert W. Doty, Victor Songmene, Fawzy H. Samuel

**Affiliations:** 1 Département des Sciences Appliquées, Université du Québec à Chicoutimi, Chicoutimi, QC G7H 2B1, Canada; gen13es@hotmail.com (E.S.); nabawyahmed2020@gmail.com (A.M.N.); fawzy-hosny.samuel@etsmtl.ca (F.H.S.); 2 Materials Technology, General Motors Global Technology Center, Warren, MI 48093-2350, USA; herb.doty@gm.com; 3 Département de Génie Mécanique, École de Technologie Supérieure, UQ, Montreal, QC H3C 1K3, Canada; victor.songmene@etsmtl.ca

**Keywords:** aluminum alloys, melt treatment, additives, heat treatment, tensile properties, Q-index

## Abstract

The present work investigated the effect of aging treatment on the microstructure and tensile properties of an Al-2%Cu base alloy containing various additions of Zr and other alloying elements. Aging was carried out at temperatures of 180–300 °C for different aging times at each temperature. The tensile properties indicated that Zr additions improved the strength of the base alloy, especially at high Zr levels at 180 °C. At the 220 °C aging temperature, however, while Zr addition did not have a beneficial effect on the alloy strength, the ductility was found to improve. Zr-Ti combined additions had a significant effect on the microstructure of the base alloy, as the morphology of the α-Al grains transformed into a non-dendritic morphology, and the grain size decreased sharply. These effects were at their maximum at 180 °C and 0.5 wt% Zr addition. Moreover, the Zr-containing alloys aged at higher temperatures, such as 220 °C and 240 °C, maintained a noticeably higher level of strength over the base alloy aged at the same temperatures. Quality index charts based on the tensile test data also reflected an improvement in alloy quality and strength with Zr-Ti combined additions.

## 1. Introduction

Aluminum alloys are extensively used in the automotive, transportation, and aerospace industries, where the need for high-strength, lightweight, fuel-efficient, and cost-effective materials is critical [1,2,3,4]. The high strength of Al-Cu alloys is promising for these applications due to the added benefit of enhanced mechanical properties following precipitation-hardening heat treatments [5,6].

Heat treatment is a process used to improve the properties of aluminum alloys through a number of microstructural changes that take place as a function of the applied heat treatment parameters of temperature and time. The most prevalent heat treatment for industrial applications is the T6 treatment. This technique involves three stages: solution heat treatment, quenching, and aging. Solution heat treatment is used to maximize the dissolution of alloying elements, particularly strengthening elements such as Mg and Cu, in solid solution to take advantage of precipitation hardening during the subsequent aging treatment. Solution treatment (or solutionizing) is carried out at a high temperature close to the solidus temperature of the alloy. The solution time must be long enough to homogenize the alloy and to ensure a satisfactory degree of precipitates in the solution. While solution treatment of Al-Si-Mg alloys can be safely carried out at 540 °C, alloys containing Cu are solution-treated at a lower temperature due to the risk of local or incipient melting of Cu-containing phases. The solution temperature of Al-Si-Cu-Mg alloys is thus restricted to 495 °C to avoid such incipient melting of the copper-rich Al_2_Cu phase.

After solutionizing, the alloy is ‘quenched’ to a lower temperature in water or any suitable quenching medium to preserve the alloying elements in the solid solution formed during the solution heat treatment. Age hardening or precipitation hardening follows thereafter, where the alloy is subjected to a specified temperature for a certain period (termed artificial aging). This allows the alloying elements to diffuse through the microstructure and form intermetallic precipitates that act as strengthening phases. The properties of the alloy depend on the aging temperature and time. Typically, the hardness and strength of the alloy initially increase with time and the size of the precipitate particles until it reaches the peak aging point, where maximum strength is obtained. With further aging, the strength and hardness decrease, or softening of the alloy occurs, corresponding to the overaging condition [7].

In the case of the Al-Cu alloys mentioned earlier, during the precipitation-hardening heat treatments, the supersaturated Al-Cu solid solution gives rise to Guinier–Preston (GP) zones and *θ*″, *θ*′, and *θ* phases, with a loss in coherency and an increase in particle size as aging time is increased. If the aging time were fixed, then this sequence of events would occur with an increase in the aging temperature [8,9,10,11].

Zirconium is one of the transition elements that interact with aluminum in a peritectic reaction to form Al_3_Zr. The Al_3_Zr phase is distinguished by two different crystallographic structures. The first is the stable, tetragonal DO_23_ structure, which is body-centered tetragonal with eight atoms per unit cell [12]. The second is the coherent metastable phase Al_3_Zr (L1_2_), which is simple cubic with four atoms per unit cell with a very small lattice mismatch with α-Al. The Al_3_Zr intermetallic structure is dependent on the Zr addition to the alloy and the cooling rate. The L1_2_-Al_3_Zr type can be formed either as a primary phase during solidification with potent nucleating efficiency for α-Al or as a hardening phase decomposed from supersaturated α-Al during solution heat treatment [13].

**Figure 1:**
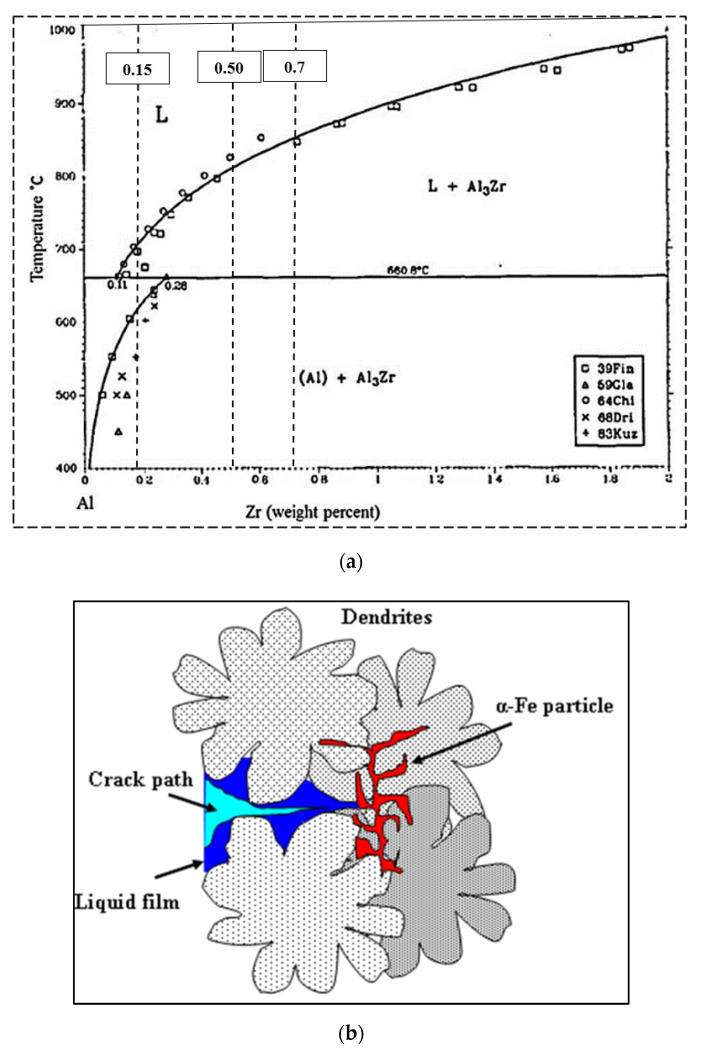
(**a**) Al-rich corner of the Al-Zr binary phase diagram (adapted from [18]). (**b**) Schematic sketch showing how α-Fe particles obstruct crack propagation in the alloy.

The effect of Zr additions to aluminum and Al alloys has been studied in combination with other additions, such as scandium, rare earth metals, etc., by various researchers [12,14,15,16]. However, there is limited literature regarding the effect of Zr alone on the strength of Al-Cu alloys [14,15,16]. The addition of Zr to aluminum alloys results in the coherent Al_3_Zr phase, which is known to bring about a refined microstructure, impede dislocation motion, and, in turn, improve the mechanical properties and hardness of the alloy [17]. According to the binary Al-Zr phase diagram shown in Figure 1a adapted from [18], dispersoid precipitation is only thermodynamically possible if the Zr concentration is higher than 0.08% at temperatures approaching 500 °C. Precipitation will thus occur at the dendrite cores with Zr concentrations of more than 0.15% and will continue until the concentration is reduced to 0.08 wt%, which is the minimum required limit for precipitation at or near 500 °C. The similarity of the structure of the L1_2_-type Al_3_M compounds to that of the Al matrix leads to a coherent interface, thereby minimizing the surface energy and maximizing the strengthening effect [19].

When a moving dislocation is hindered by a precipitate, a balance of forces will develop due to the precipitate/dislocation interaction. The force balance that develops between the moving dislocation and the precipitates can be described using the following equation [20]:

(1) $$F=2\Gamma \sin\left(\frac{\theta_{C}}{2}\right)=2\Gamma \cos\left(\frac{\Psi_{C}}{2}\right)$$

where *F* represents the precipitate resistance force, Γ indicates the line tension of the moving dislocation, and *Ψc* is the critical dislocation bowing angle. It is apparent that as the precipitate size decreases, the angle (*θc*) increases, whereas *Ψc* decreases, resulting in higher resistance force (*F*), as well as line tension of the moving dislocation (T), reflecting the presence of strong obstacles or precipitates.

The reduction in the strength and quality index values upon increasing the aging temperature is related to the formation of coarser precipitates with lower density in the matrix displaying large inter-particle spacing. These changes facilitate dislocation motion and result in softening effects, thereby reducing the strength and the quality index values of the alloy castings under investigation [6].

Drouzy et al. [21] first proposed the quality index concept in 1980 and used numerical values termed quality indices, which related the tensile properties of Al-7Si-Mg alloys to the alloy quality, as represented by the following equation:

(2) Q=σ UTS+dlog (Ef)

where *Q* is the quality index in MPa; *σ_UTS_* refers to the ultimate tensile strength in MPa; *E_f_* refers to the percentage elongation to fracture; and *d* is a material constant equal to 150 MPa for Al-7Si-Mg alloys. The probable yield strength (*σ_P(YS)_*) for the same alloy may be proposed as:

(3) $$\sigma_{P(YS)}\;=\;\;\;a\;\sigma_{UTS}\;-\;b\;\log\;(E_{f})\;+\;c$$

where the coefficients *a*, *b*, and *c* for Al-7Si-Mg were determined to be 1, 60, and −13, respectively. Equation (2) is used to generate the iso-Q lines, whereas Equation (3) is used to generate the iso-probable yield strength lines in the quality charts proposed by Drouzy et al. [21], which help to determine the most appropriate metallurgical conditions that provide the best possible compromise between tensile properties and casting quality. This concept was later extended to other alloy systems such as Al-Cu and Al-Si-Cu-Mg alloys by other researchers [22,23,24] and has been used to construct quality charts that provide the tools to predict the best conditions to achieve quality castings with the targeted tensile properties.

Caceres et al. (1999) [22] studied the effect of aging on the quality index of the A201 Al-Cu casting alloy. They observed that the tensile strength versus elongation-to-fracture plot followed a circular pattern in this case. Using an analytical model to construct the quality index chart for the A201 alloy, they proposed that the circular pattern shown by the quality index resulted from the transition from the high Q value of the underaged condition to the low Q values of the overaged condition.

In their investigations of as-cast and T6-heat-treated Al-4.5Cu-0.3Mg-0.05Ti alloy containing 0.05–0.5 wt% Zr additions, Kamali et al. (2022) [25] reported that the highest quality index value was obtained for the T6-treated 0.3 wt% Zr-containing alloy. Quality charts were also used in our study to examine the effects of alloying additions and heat treatment conditions on the resultant tensile properties.

In the present study, the effects of Zr and Ti additions/interactions to/with an Al-2%Cu base alloy (referred to in this article as alloy 220A) for automotive applications were investigated in terms of microstructure, tensile properties, and age-hardening behavior. This alloy is characterized by its high susceptibility to hot tearing during solidification. Thus, grain refining is considered one of the most crucial parameters to be considered in this context. As inferred from Figure 1a, Zr additions are considered to be low (0.15%)—which dissolve at 700 °C; medium (0.5%)—which dissolve at 800 °C; and high (0.7%)—which cannot be dissolved fully at 850 °C. In this alloy, the Mn/Fe ratio is about 1.4, and hence, the Fe-based intermetallic that would precipitate should be mainly α-Al_15_(Fe,Mn)_3_Si_2_. From an examination of the fracture behavior of the tensile-tested samples, the precipitation of the α-Fe phase proved effective in restricting crack propagation during solidification, as shown schematically in Figure 1b.

## 2. Materials and Methods

The Al-2%Cu-based alloy was received as small ingots with the composition shown in Table 1. These ingots were melted in a 35 kg capacity crucible-type furnace equipped with a degassing impeller using dry argon. The melting temperature was kept at 870 °C ± 5 °C.

At this temperature, Zr was added in different amounts, 0.3%, 0.5%, and 0.7 wt%, in the form of Al-15%Zr master alloy. The melt was held at this temperature for 25 min to allow for the maximum dissolution of Zr. During this period, the melt was mechanically stirred. The temperature was then reduced to 750 °C. Designated amounts of alloying elements were added in the form of master alloys as follows: 0.15 wt% Ti was added in the form of Al-5%Ti-1%B, and 150 ppm Sr was added in the form of Al-10%Sr. The degassed melt was poured at 750 °C ± 5 °C into a preheated (450 °C) ASTM B-108 permanent mold to produce the necessary tensile test samples. Each casting provided two test bars, with a gauge length of 70 mm and a cross-sectional diameter of 12.7 mm. For each pouring, a sample for chemical analysis was also taken.

The melt was used to prepare two series of alloy castings: the Zr group, as shown in Table 2, and the Zr-Ti group, as shown in Table 3. The Zr group was prepared from the as-received alloy—coded 220 alloy (developed by General Motors for automotive applications). This was termed 220A in our study (A for as-received). The other two alloys, 220L and 220M with Zr additions, were prepared from this alloy (L and M representing 0.3 wt% and 0.5 wt% Zr concentrations, respectively).

Another set of castings was made using a cylindrical metallic mold preheated at 450 °C. Three compositions were selected: (a) Al-2%Cu, (b) Al-2%Cu-0.3%Zr, and Al-2%Cu-0.15%Ti. The liquid metal was poured into the preheated cylindrical metallic mold. The mold body was coated with B-3 refractory material (vermiculite), whereas the casting cavity was coated with graphite. The purpose of this series was to compare the grain refining effectiveness of Zr and Ti individually.

The test bars were categorized into sixteen sets, each set containing five bars: one set was kept in the as-cast condition, and the second set was solution heat-treated at 490 °C/8 h (with a heating rate of 93 °C/h) and then quenched in warm water at 60 °C. The remaining fourteen sets were likewise solution heat-treated, quenched, and then artificially aged as follows: seven sets were aged at 180 °C for different aging times of 2, 4, 6, 10, 16, 24, and 48 h; another seven sets were aged at 220 °C for the same seven aging times. All heat treatments were carried out in a programmable Blue M forced-air furnace with a temperature control of ±2 °C. Table 4 summarizes the solution heat treatment and aging parameters that were applied to the test bars.

Tensile testing was carried out at room temperature using a Servohydraulic MTS 810 Material Testing Machine at a strain rate of 1 × 10^−4^/s. The average UTS, YS, and %El values obtained from each set of five samples were considered to be the values representing the specified condition.

For grain size measurements, rounded 1.25 cm Ø samples were cut from as-cast tensile bars and polished and etched using a solution made up of 12.5 g of CrO_3_, 2.5 mL of HF, 30 mL of HCl, 40 mL of HNO_3_, and 42.5 mL of water. The measurements were carried out employing a Clemex image analyzer in conjunction with an optical microscope and using the line intercept method.

The Brinell hardness test consists of indenting a metal surface with a steel ball, 10 mm in diameter, at a load of 500 kgf for 30 s. Brinell hardness tests were performed on the shoulders of the fractured tensile samples.

A Hitachi-SU8000 field-emission scanning electron microscope (FESEM) was used to obtain further images of the deformed tensile bar cross-sections. This instrument can provide clear and less electrostatically distorted high-resolution images even at low voltages, with an image resolution of 2.1 nm at 1 kV and 1.5 nm at 15 kV. The FESEM instrument also comes equipped with a standard secondary electron detector (SE), a backscatter electron detector (BSE), and an energy-dispersive X-ray spectrometer (EDS).

Transmission electron microscopy (TEM) was used in order to observe and identify the strengthening precipitates in heat-treated samples and to investigate the coherency of the precipitates with the matrix. An FEI Tecnai G2 F20 electron microscope was employed for this purpose, operated at an accelerating voltage of 200 kV. Specimen preparation involved cutting a thin slice from the bulk sample, from which 3 mm discs were punched out and ground to 50 μm thickness; the discs were further thinned down to 5–10 μm followed by ion milling to achieve electron transparency.

## 3. Results and Discussion

### 3.1. Zr Group of Alloys

#### 3.1.1. Microstructural Characterization

##### Low Solidification Rate

**Figure 2:**
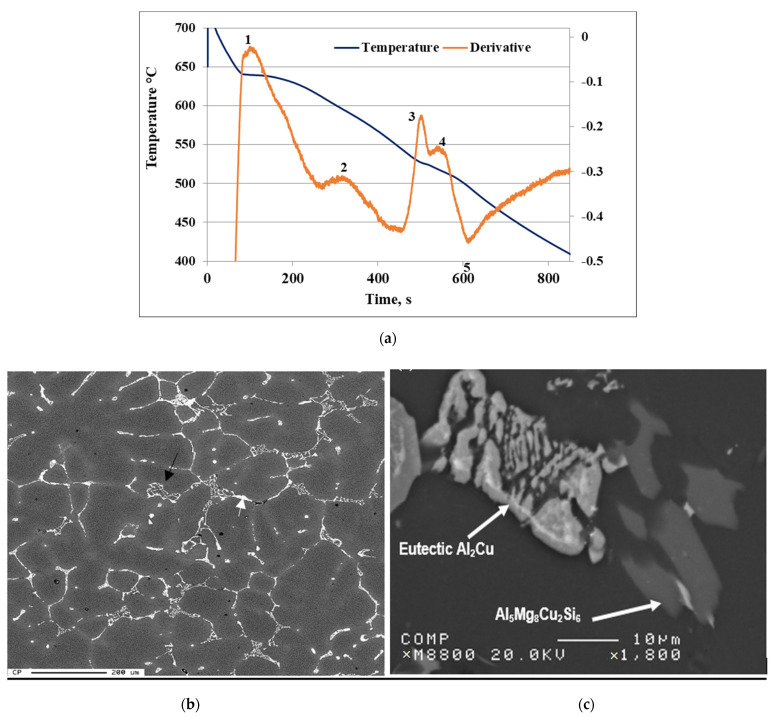

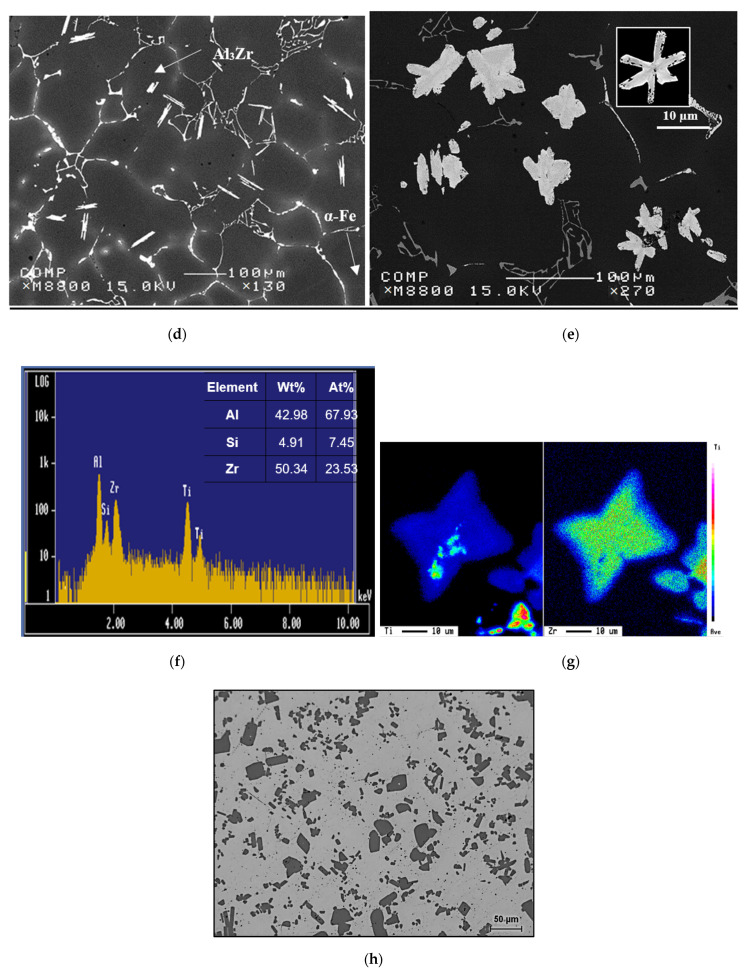
(**a**) Solidification curve and first derivative of the 220A base alloy. (**b**–**e**) SEM images showing the alloy microstructure of: (**b**,**c**) 220A, (**d**) 220L, and (**e**) 220M alloys—see the inset micrograph in (**e**) highlighting a star-like particle; (**f**) EDS spectrum corresponding to the star-like particles in (**e**); (**g**) Zr and Ti distributions in the star-like particle; (**h**) optical micrograph showing the microstructure of the Al-15% Zr master alloy used in this study.

The solidification curve and first derivative of the 220A base alloy (solidification rate ~0.8 °C/s) are displayed in Figure 2a. The alloy starts to solidify at 640 °C (reaction #1) through the development of the α-Al dendritic network, followed by the precipitation of the α-Fe (Al_15_(Mn,Fe,Cu)_3_Si_2_) phase at 619 °C (reaction #2), and then Al_2_Cu (reaction # 3) and Q-Al_5_Mg_8_Cu_2_Si_6_, which precipitate simultaneously as the last reaction at 495 °C (reaction #4). Reaction #5 represents the end of solidification at 500 °C.

Figure 2b–f display the changes in the shape and density of precipitated Al_3_Zr phase particles during solidification (at 0.8 °C/s). The microstructure of the base alloy 220A in Figure 2b displays a grain structure with Al_2_Cu (white arrow) and α-Fe (black arrow) phase particles precipitating mainly along the grain boundaries. With the addition of 0.3% Zr to the base alloy (i.e., 220L alloy), bright particles in the form of short platelets are seen all over the matrix, as depicted in Figure 2d. Increasing the Zr concentration to 0.5% resulted in the precipitation of the Al_3_Zr phase in the form of star-like shaped particles in the 220M alloy (Figure 2e). The corresponding EDS spectrum is shown in Figure 2f. It is thus reasonable to conclude that the change in the above-mentioned particle shape took place during the period when the melt temperature was reduced from 870 °C to 750 °C. Figure 2g represents the microstructure of the Al-15%Zr master alloy used in the present study. Apparently, all precipitated particles resulted from the decomposition of the master alloy during the melting process.

The above phases were also detected and analyzed using electron probe microanalysis, which revealed the same features that were observed using optical microscopy. The coarse morphology of the α-iron, Al_2_Cu, and Q-Al_5_Mg_8_Cu_2_Si_6_ phases may be attributed to the low silicon content and to the low solidification rate of 0.8 °C/s. The low Si level in the alloy contributes to the production of a larger fraction of the primary α-Al, which in turn causes the final Cu- and Fe-enriched liquid to become concentrated in a few large-sized liquid pools. The intermetallic particles will thus precipitate in these regions in the form of long interconnected particle stringers. At the same time, it was observed that the low solidification rate provides the required period for the significant growth of intermetallic particles through solidification, thereby producing coarse-sized particles. In spite of the presence of a high iron content in the base alloy, the β-iron phase did not precipitate, whereas the α-iron phase was detected. This fact may be attributed to the high Mn content in this alloy. Mn neutralizes the Fe content by promoting the formation of the α-iron Chinese script phase at the expense of the formation of the β-iron phase in platelet form.

##### High Solidification Rate 

**Figure 3:**
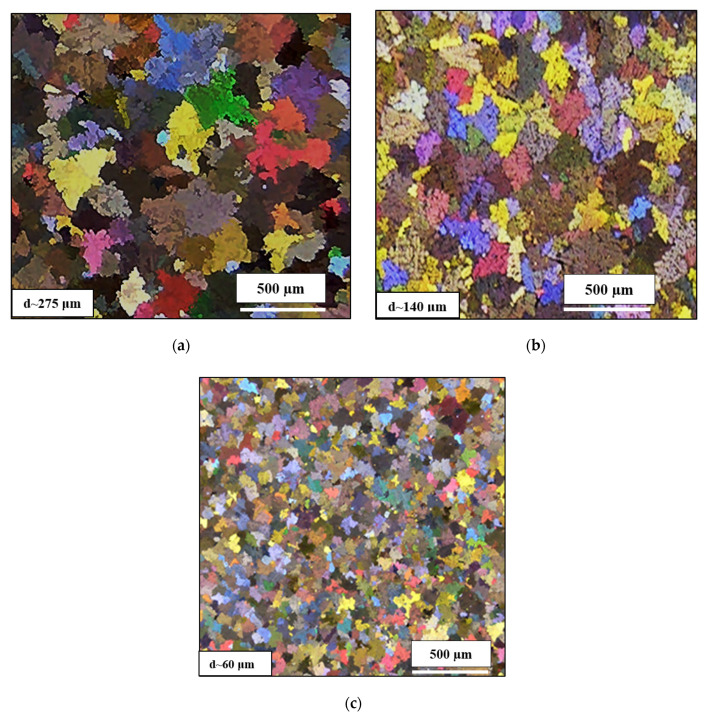
Optical macrographs and average grain sizes of the as-cast microstructure in the (**a**) 220A, (**b**) 220L and (**c**) 220M alloys.

**Figure 4:**
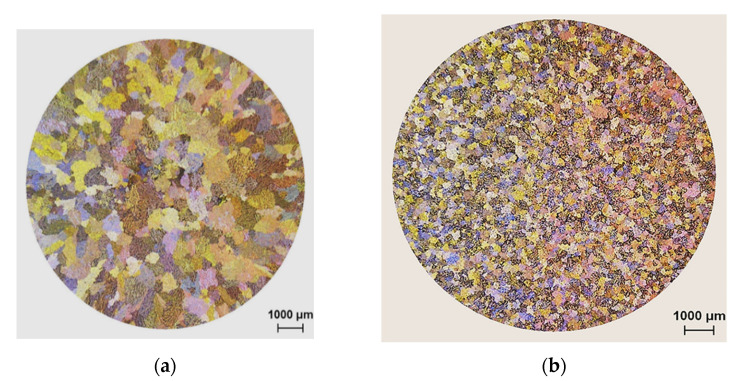

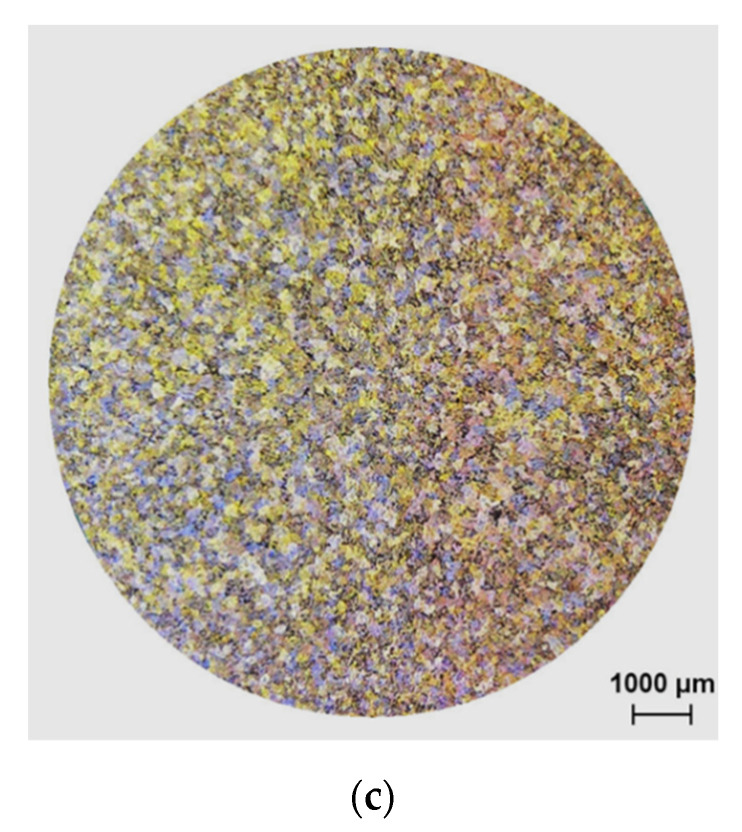
Optical macrostructures showing the grain size in experimental Al-Cu alloy with: (**a**) no Zr, (**b**) 0.3 wt% Zr, and (**c**) 0.15 wt% Ti.

Optical macrographs of the as-cast macrostructure in alloys 220A, 220L and 220M (solidification rate ~8 °C/s) are displayed in Figure 3a–c, respectively. It is evident that the grain sizes decrease from (a) to (b) to (c), i.e., with increasing Zr content. The refined macrostructure, along with the impedance of dislocation motion due to the presence of Al_3_Zr, allows the alloy samples to strain-harden to a remarkable degree before fracturing. Additionally, the addition of only 0.3 wt% Zr alters the grain structure from a columnar one (Figure 4a) to an equiaxed one (Figure 4b). The same effect was obtained with the addition of 0.15%Ti added in the form of Al-5%Ti-1%B (approximately 200 µm), as seen in Figure 4c.

Kamali et al. (2022) [25] studied the effect of 0.05–0.5 wt% Zr additions on the structure and tensile properties of an Al-4.5Cu-0.3Mg-0.05Ti (wt%) alloy solidified at a high cooling rate (18 °C/s). They reported that the as-cast structure exhibited equiaxed grains of α-Al with an average size of 64 μm, which led them to conclude that the grain size is unaffected by the Zr additions, indicating the ineffectiveness of Zr in the grain refinement of the alloy. In the present study, the samples in Figure 4 were obtained from tensile bars, i.e., at a cooling rate of about 8 °C/s, for test bars obtained from the ASTM B108 permanent mold. The grain refinement in Figure 4b is clearly obtained with 0.3 wt% Zr and comparable to that achieved with 0.15 wt% Ti, as seen in Figure 4c. In addition, in Figure 3c, the average grain size achieved in the 220M alloy (containing 0.3 wt% Zr) is 60 μm, which would indicate that, in the work of Kamali et al. [25], the higher cooling rate had a much greater influence on the grain refinement, so the effect of the Zr addition could not be separately discerned. As will be shown further on in Figure 14, the non-dendritic equiaxed nature of the grain structure of alloys G1Z and G3Z (containing 0.15%Zr and 0.3%Zr additions, respectively) is clearly noted, with the G3Z alloy exhibiting a much finer grain structure and virtually no cracks.

#### 3.1.2. Tensile Properties

**Figure 5:**
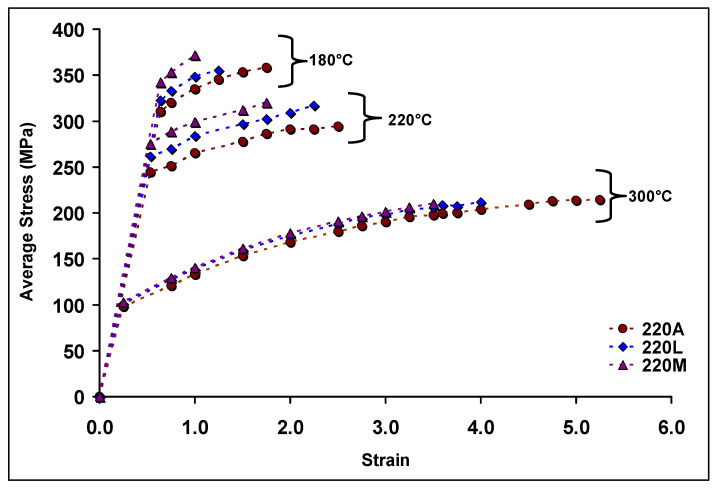
Average stress–strain curves for alloys 220A, 220L, and 220M aged at 180 °C, 220 °C, and 300 °C. All tensile bars were aged for 10 h.

Stress-strain curves for the 220A, 220L, and 220M alloys are shown in Figure 5, for alloys aged at 180 °C, 220 °C, and 300 °C. All samples were aged for 10 h at the aging temperature. Each data point represents the average of five tests in order to display the overall alloy behavior with and without Zr. The strain to fracture is seen to increase for alloys aged at higher temperatures and to decrease with increasing Zr content. Stress–strain curves at 155 °C and 200 °C reveal lower values of peak stress than those at 180 °C, therefore indicating that peak aging occurs at 180 °C.

The alloys shown in Figure 5 undergo strain hardening to fracture and do not neck. Therefore, the term ‘ultimate tensile stress’ cannot be applied here, as it implies that necking follows the point of maximum tensile stress. Moreover, given that the strain to fracture at 180 °C is very low, a comparison of the recorded tensile stresses must also be made at a very low value of strain.

**Figure 6:**
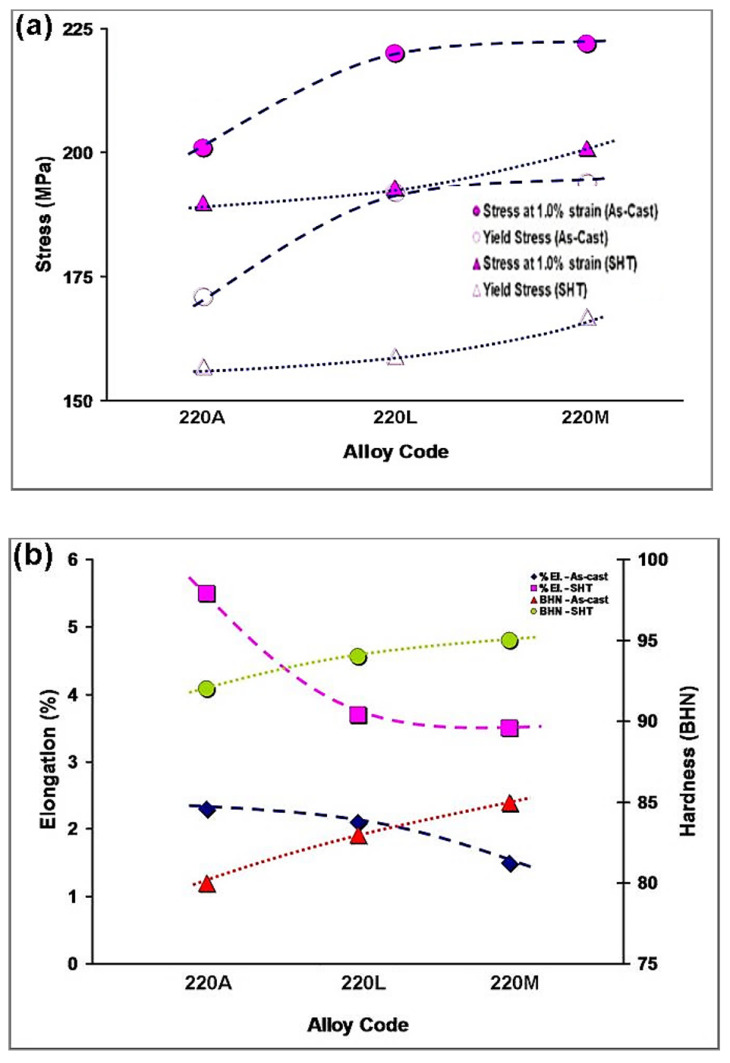
(**a**) Tensile stress (at 1.0% strain) and yield stress and (**b**) percent elongation and Brinell hardness in the as-cast and solution heat-treated (SHT) conditions.

**Figure 7:**
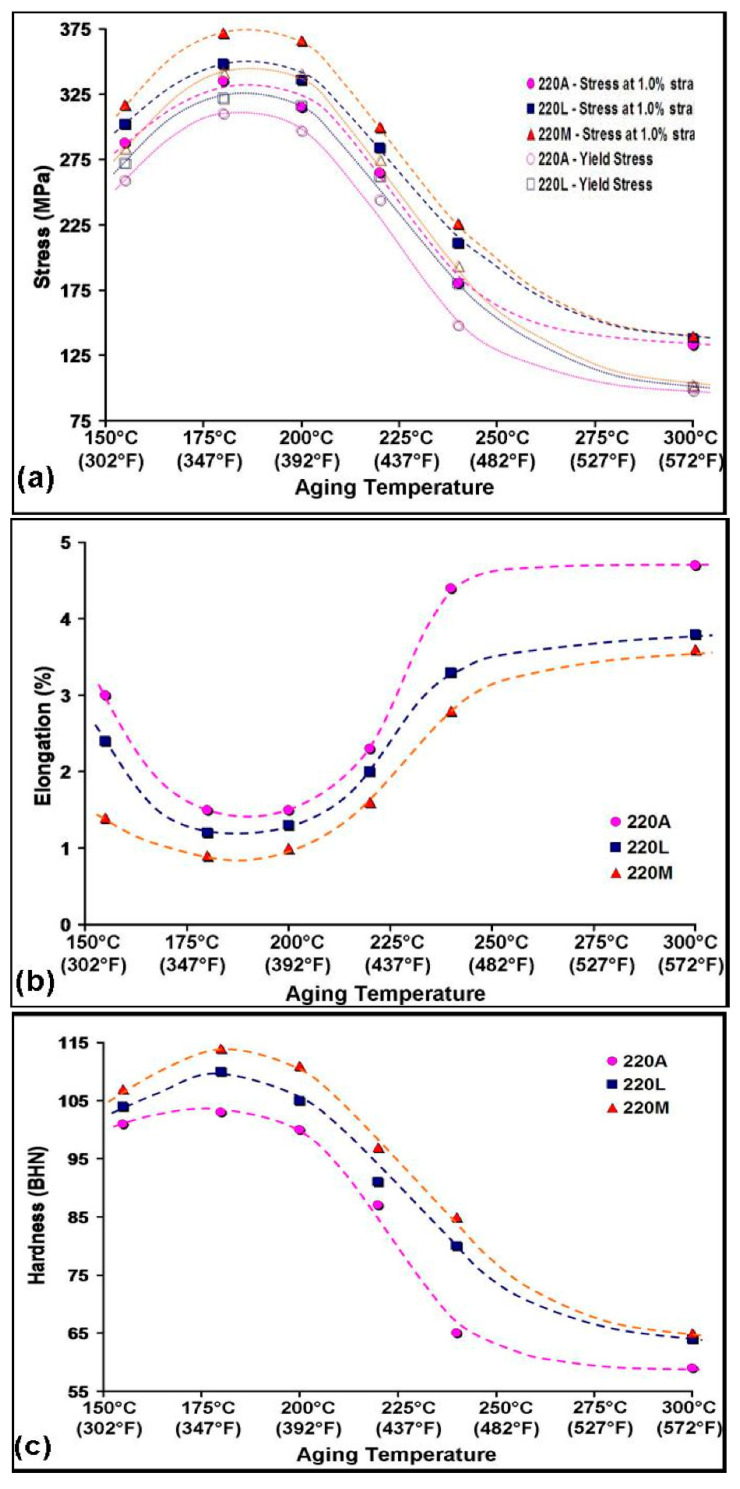
(**a**) Tensile stress (at 1.0% strain) and yield stress, (**b**) percent elongation, and (**c**) Brinell hardness at aging temperatures of 155 °C, 180 °C, 200 °C, 220 °C, 240 °C, and 300 °C.

The mechanical deformation and hardness test curves are shown in Figure 6 for the as-cast and solution heat-treated conditions and in Figure 7 for the aged conditions. From Figure 6a, an increase in tensile and, in turn, yield stress at all temperatures up to 300 °C with Zr content is observed. The drop in stress from 180 °C to 200 °C is not as gradual in the 220M alloy as it is in the 220A and 220L alloys, demonstrating a resistance to softening in the 220M alloy. There is a very pronounced decrease in stress from 180 °C to 240 °C, followed by a more gradual decline from 240 °C to 300 °C in the three alloys. Nevertheless, the Zr-containing alloys maintain higher values of stress at all temperatures until 300 °C. At 300 °C, the data points appear to converge to a common value. This is most likely attributed to the precipitates becoming coarse and incoherent with the surrounding matrix at 300 °C, thus reducing the maximum attainable stress in the alloys.

The elongation behavior with aging temperature is given in Figure 7, where the ductility decreases with Zr content due to the hardening effect of Zr. All alloys reach a minimum in elongation at 180 °C. The drop in elongation from 155 °C to 180 °C is more pronounced in the 220A and 220L alloys, whereas the decrease in elongation in the 220M alloy occurs more gradually. The increase in ductility with temperature is very gradual in the 220M alloy compared to the 220A alloy, which increases very suddenly in elongation at aging temperatures greater than 220°C. Alloys 220A and 220L reach a plateau (i.e., saturate) in the elongation value from 240°C to 300 °C, whereas the 220M alloy exhibits a steady climb in elongation. The resistance of the 220M alloy to softening (Figure 7a) from 180 °C to 200 °C is mirrored in Figure 7b in that there is very little change in ductility, i.e., a resistance to elongation, from 180 °C to 200 °C.

The Zr-containing alloys demonstrate a similar trend in hardness (Figure 7c) from 155 °C to 300 °C and converge to nearly the same value at 300 °C. On the other hand, the hardness values for the base 220A alloy fall below those of the Zr-containing alloys at every aging temperature. The behavior observed in the 220A alloy at 155 °C to 220 °C is similar to the 220L and 220M alloys but at lower hardness values. Subsequently, the rate of softening in the 220A alloy increases rapidly after 220 °C, resulting in a sudden drop in hardness from 220 °C to 240 °C. The further decrease in hardness in the 220A alloy from 240 °C to 300 °C occurs more gradually. The peak hardness in all alloys is observed at 180 °C.

**Figure 8:**
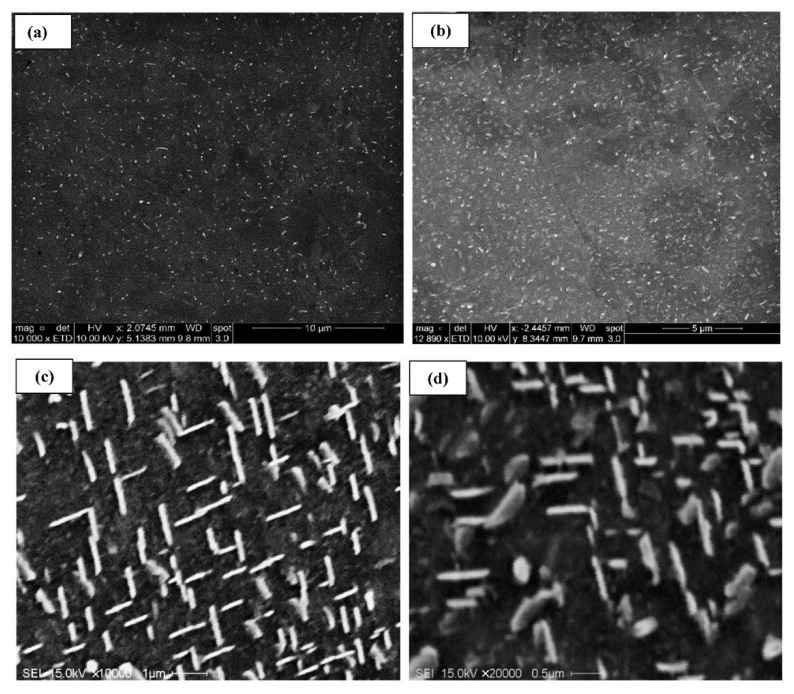
Electron images showing precipitation observed in alloy 220A after aging at (**a**) 180 °C—at 10,000×; (**b**) 220 °C—at 10,000×; (**c**) 300 °C—at 10,000×; and (**d**) 300 °C—at 20,000× magnification.

**Figure 9:**
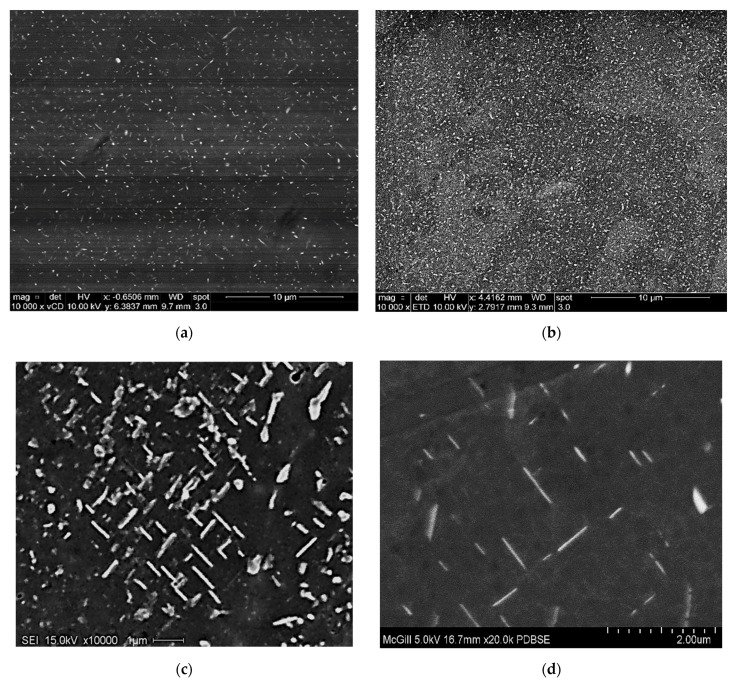
Electron images showing precipitation observed in alloy 220M at (**a**) 180 °C—at 10,000×; (**b**) 220 °C—at 10,000×; (**c**) 300 °C—at 10,000×; and (**d**) 300 °C—at 20,000× magnification.

In this section, all micrographs were taken at 5 kV except for Figure 8b, which was taken at 2 kV to verify the sphericity of the precipitated particles. Figure 8 and Figure 9 demonstrate the precipitation sequence observed in alloys 220A and 220M, respectively. It can be seen that at 180°C, the regions of both alloys are governed by the presence of GP zones (Figure 8a and Figure 9a), which coincides with the observed peak values in tensile stress and hardness for this aging temperature. This gives way to a mixture of *θ”* and *θ’* precipitates at 220 °C (Figure 8b and Figure 9b), the particles of which begin to coarsen in size all the way up to 300 °C. Figure 8c and Figure 9c represent the point at which coarsening occurs by a mixture of *θ*′ and *θ*. Additionally, the morphology of the precipitates changes to a rod-like shape at 300 °C.

These SEM micrographs (Figure 8 and Figure 9), however, are very similar from alloy to alloy at a given temperature. Therefore, the effect of Zr on the base alloy in terms of the formation of Al_3_Zr particles is difficult to explain based on these images alone. The sole means of indication that there is a Zr strengthening effect is the mechanical testing curves (Figure 5, Figure 6 and Figure 7). Additionally, the alloy chemistry presents the question of which precipitates are at play at each temperature. Figure 8c—as well as Figure 8a—clearly demonstrates a region of peak aging (T = 180 °C), underaging, and overaging. Therefore, a change in the particle form to coarser and semicoherent at 220 °C would explain the drop in stress and hardness compared that at to 180 °C. However, the 220 A alloy still maintains values of stress and hardness lower than either of the Zr-containing alloys. Transmission electron microscopy work and a more thorough analysis are required to determine which precipitates are at play in these alloys, as this cannot be solely explained based on SEM micrographs.

### 3.2. Zr-Ti Group of Alloys

**Figure 10:**
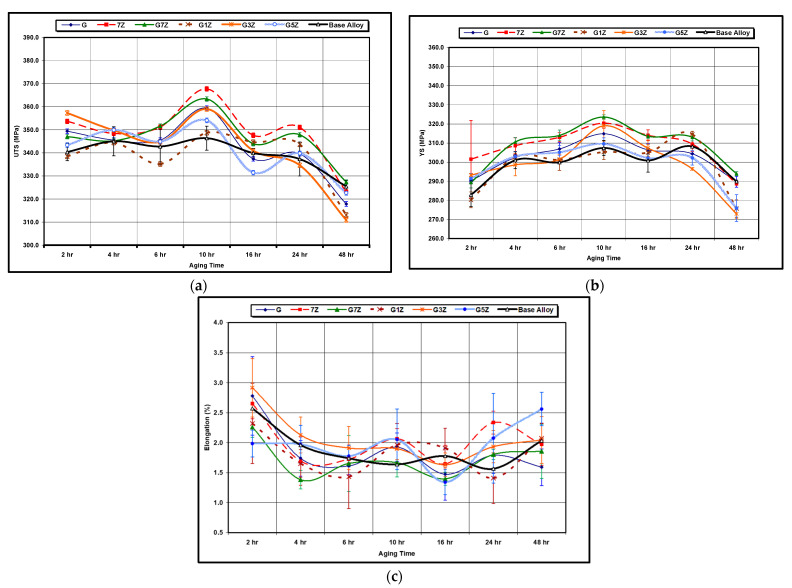
Effect of aging time on the tensile properties of the Zr-Ti alloy group at 180 °C aging temperature: (**a**) UTS, (**b**) YS, (**c**) %El.

Figure 10 illustrates the effects of various Zr-Ti additions on the tensile properties after different aging times, between 2 h and 48 h, at an aging temperature of 180 °C. It can be observed that there are two age-hardening peaks occurring at 10 h and 24 h. In addition, the tensile strength and yield strength improve as the Zr concentration level increases, with their values increasing from 346 MPa to 368 MPa and from 307 MPa to 324 MPa, respectively, as the Zr content increases from 0 wt% to 0.7 wt%; the ductility, however, is not greatly improved by further additions of Zr. Again, it should also be noted that there is no observable difference in strength as the Ti increases from 0.02 wt% Ti to 0.15 wt% Ti for 0.7 wt% Zr alloys, whereas the ductility is better at lower additions of Ti. There is a drop observed in tensile strength and yield strength values as aging time increases to 48 h, and at the same time, the ductility is seen to increase; this may be attributed to the overaging process.

In the Al-Cu-Mg-Si system, the main hardening phases to be expected are θ′(Al_2_Cu), β″(Mg_2_Si), Q′(AlMgSiCu), S″(Al_2_CuMg), and the Si phase [26,27]. Thus, the predicted phases for the alloy investigated here (which has the composition Al-2Cu-1Si-0.4Mg-0.42Fe-0.6Mn) are connected to said phases. It was also postulated that the Mg:Si ratio and the copper content determine the main hardening phases in the aging process [28]. With regard to the alloy under investigation, therefore, which has a Mg:Si ratio of 0.4 and a 2% Cu content, the hardening phases expected are θ′(Al_2_Cu), β″(Mg_2_Si), the Si phase, and possibly S′(Al_2_CuMg).

It is suggested that these phases and the Zr content are responsible for the two age-hardening peaks observed in Figure 10. Regarding the tensile and yield strength results presented in Figure 10, an increase in strength may be observed with an increase in Zr content from 0.3 wt% to 0.7 wt%. This increase in strength may be attributed to an increase in dislocation movement resistance resulting from various factors, such as: (a) an increase in the size of the Al_3_Zr dispersoids during aging could lead to a transition in the dislocation movement mechanism from cutting the Al_3_Zr dispersoids to bypassing them, thereby increasing the dislocation movement resistance; (b) the action of Al_3_Zr precipitates as nucleation sites for age-hardening phases would also increase the resistance to dislocation movement, as reported in a number of studies [29,30,31]; (c) it is also possible that Zr modifies the S’-phase precipitates due to the high binding energy (0.24 ± 0.02 eV) between Zr atoms and vacancies in aluminum, as mention in other investigations [32,33,34].

Based on the results of this study, the second and third factors may be considered acceptable/rational explanations for the observed increase in strength with the increase in Zr content but not so for the first factor, given that the strength decreases after 10 h of aging. There is also the possibility that such an improvement may be related to a reduction in grain size, as is discussed further in this subsection with respect to the microstructure.

**Figure 11:**
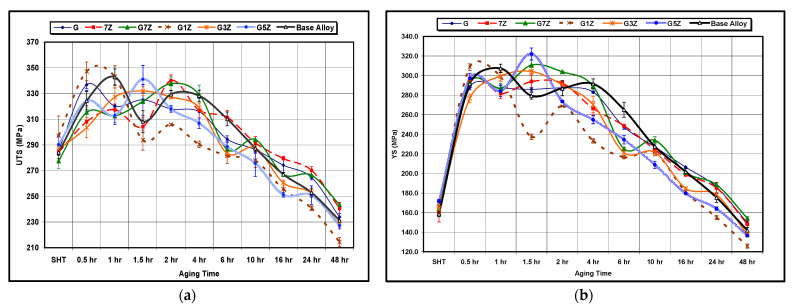

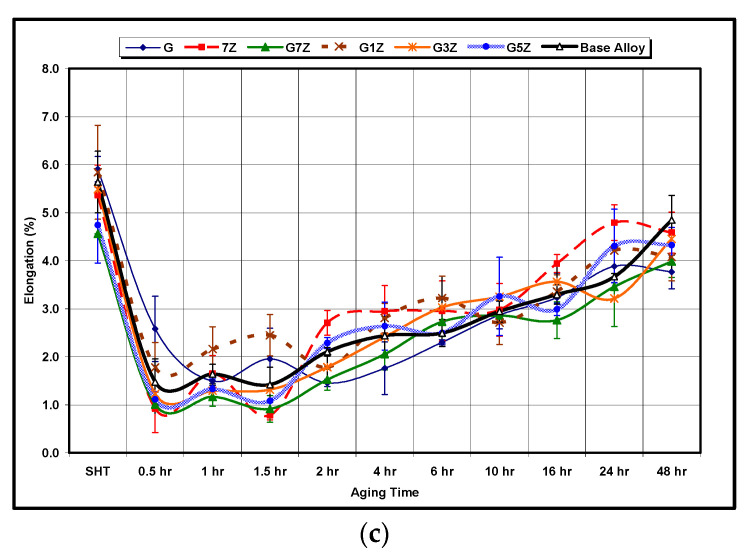
Effect of aging time on the tensile properties of the Zr-Ti alloy group at 220 °C aging temperature: (**a**) UTS, (**b**) YS, (**c**) %El.

Figure 11 shows the effects of a higher aging temperature (220 °C) on the tensile properties of the various alloy compositions for aging times ranging from 2 h to 48 h. The tensile strength and yield strength both decrease with the increase in aging time beyond 2 h. The addition of Zr does not appear to show any beneficial effects at the 220 °C aging temperature, and the alloys begin to lose strength in a noticeable way as the aging time increases beyond 2 h. This loss in strength is attributed to the high growth rate of precipitates, ultimately leading to the formation of coarse equilibrium phases of Al_2_Cu and Mg_2_Si, which is the main reason for the loss of strength. Although the alloys are seen to lose strength as the aging time increases, the elongation is found to increase, as, for example, in the case of 7Z alloy (0.7%Zr), where, after 24 h of aging, the elongation attained is 4.8% (compared to 2.7% after 2 h of aging).

A temperature of 220 °C is considered to be an elevated aging temperature at which precipitation would not occur in the normal sequences; in the case of Al-Cu systems at a high aging temperature of 220 °C, the GP zones are not expected to form because such an aging temperature would be above their solvus line, although, instead, the θ″ will nucleate directly. At the high aging temperature of 220 °C, the kinetics of the aging process are high as a result of the higher solute diffusivity [35,36], leading to peak strength in a short time, thus explaining the increase in the strength values after only 0.5 h of aging.

From the point of view of precipitation reactions, the Al-2 wt%Cu base alloy, having the chemical composition of Al-2 wt%Cu-1 wt%Si-0.4 wt%Mg-0.42 wt%Fe-0.6 wt%Mn, is considered to be a complicated system as a result of the wide variety of the age-hardening phases that are expected to form during the age-hardening treatment. As mentioned previously, the age-hardening phases under discussion are θ′(Al_2_Cu), β″(Mg_2_Si), the Si phase, and possibly S′(Al_2_CuMg); these phases have different precipitation sequences, which may occur either simultaneously or independently. As was indicated in the discussion on the effects of the 180 °C aging temperature, the Zr content has an observable effect on the precipitation process from a number of aspects; the Al_3_Zr dispersoids provide heterogeneous nucleation sites for age-hardening phases, while Zr modifies the metastable S′ phase [36,37,38,39,40]. The Zr additions produce a refined non-dendritic structure, which in turn reduces the porosity level and the amount of undissolved Al_2_Cu, thus improving alloy strength.

The quality of aluminum-based casting alloys is considered to be a key factor in selecting an alloy casting for a particular engineering application. Deciding upon the right alloy quality thus involves reaching a suitable compromise between numerous factors so as to present the least possible risk with maximum performance in combination with adequate cost-effectiveness. Alloy composition, solidification rate, heat treatment procedures, casting defects, and such microstructural features as grain size and intermetallic phases are all parameters that strongly affect alloy quality since they also influence the mechanical properties of the casting.

**Figure 12:**
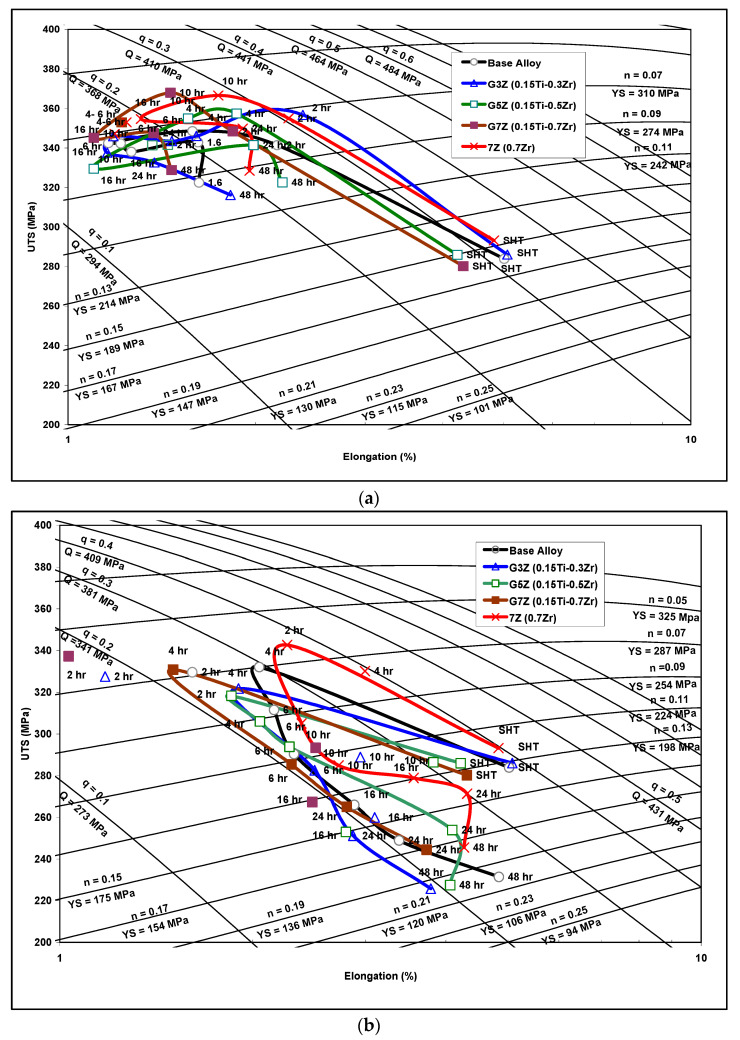
Quality index charts for Zr-Ti alloys at: (**a**) 180 °C and (**b**) 220 °C aging temperatures, showing the variation in alloy quality with aging time. Note the segregation of most of the points in (**a**) in the range of 330–400 MPa.

Figure 12 provides the quality index chart of the base alloy with various additions of Zr-Ti for different aging times at aging temperatures of 180 °C and 220 °C. Figure 12a reveals that the G3Z alloy (containing 0.15% Ti and 0.3% Zr) exhibits the highest alloy quality, with a tensile strength of 360 MPa and a Q value of about 415 MPa. Kamali et al. [25] also reported that the highest Q value for their T6-heat-treated Al-4.5Cu-0.3 Mg-0.05Ti alloy was observed with a 0.3 wt% Zr addition (249 MPa).

The plot of UTS versus %El in Figure 12b indicates that the quality behavior of Zr-Ti alloys at an aging temperature of 220 °C may be divided into two stages with two different Q-values. In each stage, the Q-values remain approximately constant by varying strength against ductility. In the first stage, the strength increases at the expense of the ductility, whereas, in the second stage, the ductility increases at the expense of the strength. This quality behavior may be described as follows: In the first few hours of aging, peak aging is reached and is associated with an improvement in strength and a reduction in ductility. Subsequently, as the overaging process is activated, the ductility increases, and the strength diminishes, thereby attaining the second stage of quality value behavior. Very little deviation from this behavior is possible because of the chemical composition and variations in the microstructure of each alloy.

**Figure 13:**
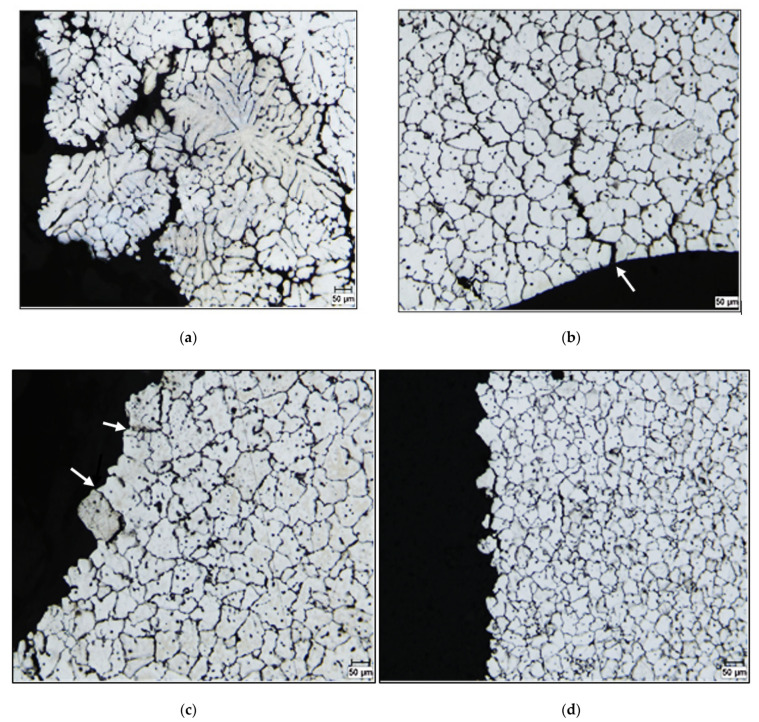
Grain structures obtained for various alloys in the Zr-Ti group: (**a**) base alloy, (**b**) G alloy, (**c**) G1Z alloy, and (**d**) G3Z alloy. Note the fine cracks in (**c**)—white arrows.

**Figure 14:**
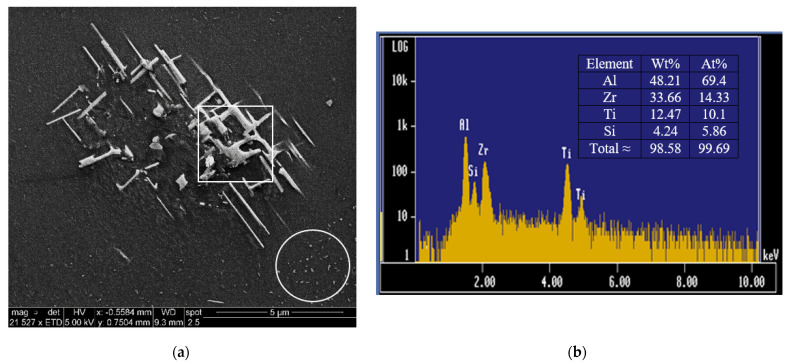
Precipitation of Zr-rich particles observed in G3Z alloy: (**a**) backscattered electron image, (**b**) EDS spectrum corresponding to the square area in (**a**). Also note the fine precipitation within the circled area in (**a**).

Figure 13 highlights the importance of the addition of the right amount of Zr to prevent hot tearing of the 220 alloy. As can be seen, the addition of 0.18%Ti or 0.15%Zr + 0.13%Ti is not sufficient to entirely remove the formation of fine cracks during solidification. Thus, the right amount of Zr is approximately 0.3% or higher. Figure 14 depicts the precipitation of Zr-rich phase particles in G3Z alloy in the form of a cluster of short rods (Figure 14a). The EDS spectrum displayed in Figure 14b shows that the composition of this phase is approximately Al_2_._9_(Zr_0_._59_Ti_0_._41_)Si_0_._24_ or Al_3_(Zr,Ti) (due to the thinness of the precipitates). Fine precipitates were also observed in the microstructure, as shown by the circled area in Figure 14a.

**Figure 15:**
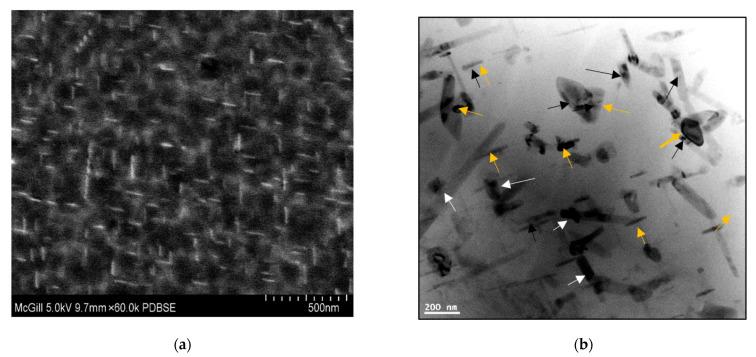

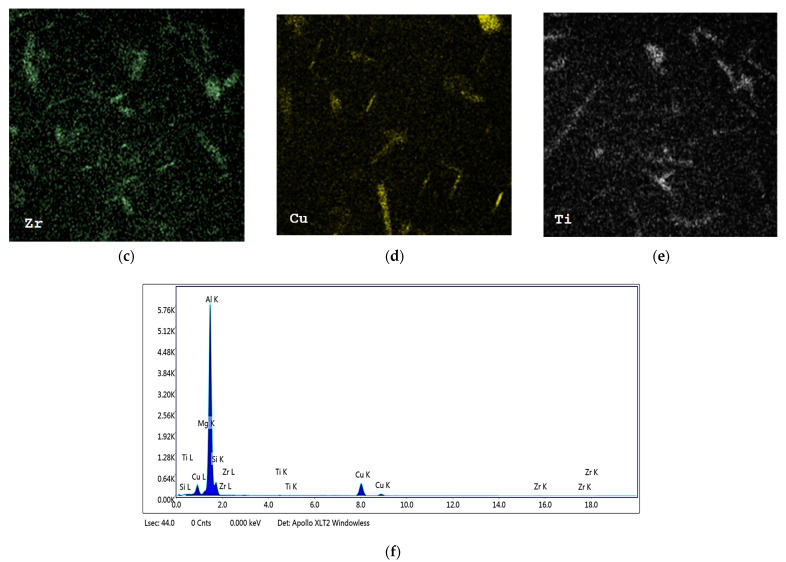
TEM bright-field electron micrographs of 354 alloy containing 0.39%Zr and 0.17%Ti and aged at (**a**) 155 °C/100 h—at 60,000× magnification; (**b**) 220 °C/100 h—at 150,000× magnification; (**c**–**e**) X-ray images of (**c**) Zr, (**d**) Cu, and (**e**) Ti distribution in (**b**); (**f**) EDS spectrum corresponding to (**b**). Black arrows point to Ti-containing phase, white arrows point to Al_2_Cu phase, and orange arrows point to Al_3_Zr phase in (**b**).

Figure 15 gives examples of the precipitation of Al_2_Cu (Figure 15a) when 354 alloy containing 0.39% Zr was aged at 155 °C/100 h and a mixture of Al_2_Cu (white arrows) and Al_3_Zr (orange arrows) when the alloy was overaged (250 °C/100 h) (Figure 15b). In both cases, the precipitated particles were in the form of short rods at two perpendicular angles similar to those presented in Figure 8c and Figure 9c [41]. It is inferred from these observations that Si has no major role in controlling the precipitation of the Al_3_Zr phase during artificial aging. Thus, it is reasonable to assume that similar reactions will take place in the present alloys. The elongated nature of the Al_2_Cu particles observed in Figure 15b,d was also observed by Kamali et al. [25] in their work.

## 4. Conclusions

1. Additions of 0.3 and 0.5 wt% Zr to the Al-2%Cu alloy lead to (a) a change in the grain structure from columnar to equiaxed throughout the casting and (b) a significant reduction in the grain size (from 275 μm to 140 μm with 0.3 wt% Zr and to 65 μm with 0.5 wt% Zr additions, respectively).
2. In the as-cast and solution heat-treated conditions, there is an increase in the tensile stress and hardness with Zr content. In contrast, elongation is seen to decrease.
3. Peak aging occurs at 180 °C, followed by softening with the increase in aging temperature. The addition of Zr reduces the softening behavior of the 220A alloy, especially at T > 220 °C, as noted for alloy 220 M from the hardness tests. Alloy 220A exhibits a dramatic reduction in hardness, coupled with a pronounced increase in ductility, after 220 °C.
4. The Zr-containing alloys are less ductile than the 220A alloy. With the increase in temperature, the tensile samples can attain higher strains, yet they suffer from a decrease in strength and hardness. The highest strength/hardness and, in turn, lowest values of ductility were all observed at 180 °C, corresponding to the peak aging condition.
5. During aging, precipitation takes place by the formation of GP zones at 180 °C, a mixture of *θ*″ and *θ*′ at 220 °C, and a mixture of *θ*′ and *θ* at 300 °C. There is a noticeable increase in stress and hardness in the Zr-containing alloys at 220 °C and 240 °C, despite the coarsening of the precipitates at 220 °C. At 300 °C, there is a change in the precipitate morphology to a rod-like shape.
6. The strength of the base alloy increases as the Zr content increases from 0.3% to 0.7% Zr after 10 h of aging at 180 °C. This increase may be ascribed to an increase in the density of Al_3_Zr particles, which act as heterogeneous nucleation sites for age-hardening phases, as well as to the modifying action of Zr on the S′ (i.e., Al_2_CuMg) phase.
7. Combined additions of Zr-Ti transform the morphology of the microstructure of the base alloy from dendritic into a non-dendritic one. In addition, the regularity of the transformation is observed to increase as the Zr content increases at the higher Ti level of 0.15%.
8. The quality of the base alloy is improved with the combined addition of Zr-Ti, for which the highest quality index in the Zr-Ti group of alloys is exhibited by the G3Z alloy (0.15%Ti-0.3%Zr) with a Q value of 420 MPa.

In conclusion, therefore, by understanding the effects of heat treatment and Zr and Ti additions on the precipitates formed in the 220-type alloys investigated, using the experimental procedures employed in this study, the parameters involved may be suitably selected to achieve the alloy composition/properties required for a specific application.

## Figures and Tables

**Table 1:** Chemical composition of the as-received alloy.

Element	Cu	Si	Fe	Mn	Mg	Ti	Al
wt%	2.00	1.05	0.42	0.60	0.40	0.02	Bal.

**Table 2:** Codes and chemical compositions of the Zr group of alloys.

220A (Base alloy)	2.00 wt% Cu, 1.05 wt% Si, 0.42 wt% Fe, 0.60 wt% Mn, 0.40 wt% Mg, 0.02 wt% Ti, Al balance
220L	220 A + 0.30 wt% Zr
220M	220 A + 0.50 wt% Zr

**Table 3:** Codes and chemical compositions of the Zr-Ti group of alloys.

Alloys		Element (wt%)							
		Cu	Si	Fe	Mn	Mg	Ti	Zr	Al
Base alloy		1.91	1.03	0.57	0.58	0.34	0.02	-	Bal.
Zr and Ti Group	G	1.85	099	0.55	0.54	0.31	0.18	-	Bal.
	G1Z	2.06	1.15	0.52	0.57	0.35	0.13	0.14	Bal.
	G3Z	2.07	1.11	0.51	0.57	0.34	0.14	0.26	Bal.
	G5Z	2.08	1.10	0.51	0.58	0.34	0.11	0.47	Bal.
	7Z	1.83	0.97	0.54	0.57	0.32	0.02	0.58	Bal.
	G7Z	1.80	0.90	0.47	0.51	0.30	0.20	0.71	Bal.

**Table 4:** Heat treatment conditions applied to tensile test bars.

Condition	Temperature (°C)	Time (h)
As-cast	N/A	N/A
Solution heat treatment	490 °C	8
Aging	180 °C	2, 4, 6, 10, 16, 24, 48
	220 °C	2, 4, 6, 10, 16, 24, 48

## Data Availability

Data are available upon request.

## References

[1] Mao X., Yi Y.-P., He H., Huang S.-Q., Guo W. (2020). Second phase particles and mechanical properties of 2219 aluminum alloys processed by an improved ring manufacturing process. Mater. Sci. Eng. A.

[2] Gao Y.H., Cao L.F., Yang C., Zhang J.Y., Liu G., Sun J. (2019). Co-stabilization of θ′-Al_2_Cu and Al_3_Sc precipitates in Sc-microalloyed Al–Cu alloy with enhanced creep resistance. Mater. Today Nano.

[3] Wang S.C., Starink M.J. (2007). Two types of S phase precipitates in Al-Cu-Mg alloys. Acta. Mater..

[4] Chen J., Deng Y., Guo X. (2018). Revisit the stress-orienting effect of θ′ in Al-Cu single crystal during stress aging. Mater. Charact..

[5] Shower P., Morris J., Shin D., Radhakrishnan B., Poplawsky J., Shyam A. (2019). Mechanisms for stabilizing θ′(Al_2_Cu) precipitates at elevated temperatures investigated with phase field modeling. Materialia.

[6] Alyaldin L., Abdelaziz M.H., Samuel A.M., Doty H.W., Samuel F.H. (2021). Effect of Transition Metals Addition on Tensile Properties of Al–Si–Cu-Based Alloys at 25 °C and 250 °C: Role of Heat Treatment. Int. J. Metalcast..

[7] Mohamed A.M.A., Samuel F.H. (2012). A Review on the Heat Treatment of Al-SiCu/Mg Casting Alloys. Heat Treatment.

[8] Guo Z., Zhao G., Chen X.G. (2015). Effects of two-step homogenization on precipitation behavior of Al_3_Zr dispersoids and recrystallization resistance in 7150 aluminum alloy. Mater. Charact..

[9] Garza-Elizondo G.H., Elsharkawi E.A., Samuel A.M., Doty H.W., Samuel F.H. (2021). Effects of Alloying Elements Additions on Ambient Temperature Performance of Al–Si–Cu–Mg Base Alloys. Int. J. Metalcast..

[10] Mikhaylovskaya A.V., Mochugovskiy A.G., Levchenko V.S., Tabachkova N.Y., Mufalo W., Portnoy V.K. (2018). Precipitation behavior of L1_2_Al_3_Zr phase in Al-Mg-Zr alloy. Mater. Charact..

[11] Abdelaziz M.H., Elsharkawi E.A., Doty H.W., Samuel F.H. (2020). Mechanical Performance of Zr-Containing 354-Type Al-Si-Cu-Mg Cast Alloy: Role of Geometrical Discontinuities. J. Mater. Eng. Perform..

[12] Zou L., Pan Q.-L., He Y.-B., Wang C.-Z., Liang W.-J. (2007). Effect of minor Sc and Zr addition on microstructures and mechanical properties of Al-Zn-Mg-Cu alloys. Trans. Nonferrous Met. Soc. China.

[13] Hernandez-Sandoval J., Abdelaziz M.H., Samuel A.M., Doty H.W., Samuel F.H., Dobrzański L.A. (2020). Effect of Zr Addition and Aging Treatment on the Tensile Properties of Al-Si-Cu-Mg Cast Alloys, Chapter 3. Advanced Aluminium Composites and Alloys.

[14] Ibrahim A.I., Elgallad E.M., Samuel A.M., Doty H.W., Samuel F.H. (2018). Effects of Addition of Transition Metals on Intermetallic Precipitation in Al–2%Cu–1%Si-Based Alloys. Int. J. Metalcast..

[15] Ding L., Zhao M., Jia Z., Weng Y., Xiang K., Wu X., Liu Q. (2021). On the formation of anti-phase boundaries and interphase boundaries in Al3Zr precipitates of Al-Cu-Zr alloy studied at atomic scale. J. Alloys Compd..

[16] Girgis A., Samuel A.M., Doty H.W., Valtierra S., Samuel F.H. (2019). On the Elevated Temperature Tensile Properties of Al-Cu Cast Alloys: Role of Heat Treatment. Adv. Mater. Sci. Eng..

[17] Abdelaziz M.H., Doty H.W., Valtierra S., Samuel F.H. (2018). Mechanical Performance of Zr-Containing 354-Type Al-Si-Cu-Mg Cast Alloy: Role of Additions and Heat Treatment. Adv. Mater. Sci. Eng..

[18] Hansen M., Anderko K. (1958). Constitution of Binary Alloys.

[19] Knipling K.E., Dunand D.C., Seidman D.N. (2006). Criteria for developing castable, creep-resistant aluminum-based alloys—A review. Z. Metallkunde.

[20] Colley L.J. (2011). Microstructure-Property Models for Heat Treatment of A356 Aluminum Alloy. Ph.D. Thesis.

[21] Drouzy M., Jacob S., Richard M. (1980). Interpretation of Tensile Results by Means of Quality Index and Probable Yield Strength-Application to Al-Si7 Mg Foundry Alloys. Int. Cast Metals J..

[22] Cáceres C.H., Din T., Rashid A.K.M.B., Campbell J. (1999). Effect of aging on quality index of an Al–Cu casting alloy. Mater. Sci. Technol..

[23] Ammar H.R., Samuel A.M., Samuel F.H., Simielli E., Sigworth G.K., Lin J.C. (2012). Influence of aging parameters on the tensile properties and quality index of Al–9 Pct Si–1.8 Pct Cu–0.5 Pct Mg 354-type casting alloys. Metall. Mater. Trans. A.

[24] Alexopoulos N.D. (2007). Generation of quality maps to support material selection by exploiting the quality indices concept of cast aluminum alloys. Mater. Des..

[25] Kamali H., Kamali E., Emamy M. (2022). Effects of Zr additions on structure and tensile properties of an Al-4.5Cu-0.3Mg-0.05Ti (wt.%) alloy. China Foundry.

[26] Wang P.-S., Lee S.-L., Lin J.-C., Jahn M.-T. (2000). Effects of Solution Temperature on Mechanical Properties of 319.0 Aluminum Casting Alloys Containing Trace Beryllium. J. Mater. Res..

[27] Apelian D., Shivkumar S., Sigworth G. (1989). Fundamental Aspect of Heat Treatment of Cast Al-Si-Mg Alloys. AFS Trans..

[28] Tillova E., Panuskova M. (2008). Effect of Solution Treatment on Intermetallic Phases Morphology in AlSi_9_Cu_3_ Cast Alloy. Metalurgija.

[29] Ghosh G., Vaynman S., Asta M., Fine M.E. (2007). Stability and Elastic Properties of L_12_-(Al,Cu)_3_(Ti,Zr) Phases: Ab initio Calculations and Experiments. Intermetallics.

[30] Knipling K.E., Dunand D.C., Seidman D.N. (2008). Precipitation evolution in Al–Zr and Al–Zr–Ti alloys during aging at 450–600 °C. Acta Mater..

[31] Kasprzak W., Amirkhiz B.S., Niewczas M. (2014). Structure and properties of cast Al–Si based alloy with Zr–V–Ti additions and its evaluation of high temperature performance. J. Alloys Compd..

[32] Li S.S., Li L., Han J., Wang C.-T., Xiao Y.-Q., Jian X.-D., Qian P., Su Y.-J. (2020). First-Principles study on the nucleation of precipitates in ternary Al alloys doped with Sc, Li, Zr, and Ti elements. Appl. Surf. Sci..

[33] Souza P.H.L., Silva de Oliveira C.A., do Vale Quaresma J.M. (2018). Precipitation hardening in dilute Al–Zr alloys. J. Mater. Res. Technol..

[34] Du J., Wen B., Melnik R., Kawazoe Y. (2015). Cluster characteristics and physical properties of binary Al–Zr intermetallic compounds from first principles studies. Comput. Mater. Sci..

[35] Ringer S.P., Hono K. (2000). Microstructural Evolution and Age Hardening in Aluminum Alloys: Atom Probe Field-Ion Microscopy and Transmission Electron Microscopy Studies. Mater. Charact..

[36] Abe T., Miyazaki K., Hirano K. (1989). Imaging of Pure Al and Aged Al-4 wt.% Cu Alloys by Field Ion Microscopy. Acta Metall..

[37] Eskin D.G. (2003). Decomposition of Supersaturated Solid Solution in Al-Cu-Mg-Si Alloys. J. Mater. Sci..

[38] Zeren M. (2005). Effect of Copper and Silicon Content on Mechanical Properties in Al-Cu-Si-Mg Alloy. J. Mater. Processing Technol..

[39] Li Y.J., Brusethaug S., Olsen A. (2006). Influence of Cu on the Tensile Properties and Precipitation Behavior of AlSi7Mg0.5 Alloy during Aging Treatment. Scr. Mater..

[40] Guiqing W., Qingzhou S. (2007). Influence of Cu Content on Ageing Behavior of AlSiMgCu Cast Alloys. Mater. Des..

[41] Samuel F.H.

